# Facile Synthesis of Non-Noble CuFeCo/C Catalysts with High Stability for ORR in PEMFC

**DOI:** 10.3390/ma18122826

**Published:** 2025-06-16

**Authors:** Ruixia Chu, Hongtao Zhang, Fangyuan Qiu, Wenjun Fu, Wanyou Huang, Runze Li, Zhenyu Li, Xiaoyue Jin, Yan Wang

**Affiliations:** 1 Automotive Engineering College, Shandong Jiaotong University, Jinan 250357, China; churx_xmu@163.com (R.C.); zht13293907672@163.com (H.Z.); fuwj@sdjtu.edu.cn (W.F.); huangwanyou2005@163.com (W.H.); 15306477529@163.com (Z.L.); 15834185062@163.com (X.J.); sdzbgqwangyan@163.com (Y.W.); 2 Intelligent Testing and High-End Equipment of Automotive Power Systems, Shandong Province Engineering Research Center, Jinan 250357, China; 3 College of Mechanical and Transportation Engineering, China University of Petroleum, Beijing 102249, China; lirunze0627@163.com

**Keywords:** CuFeCo/C, non-noble, catalyst, ORR, stability

## Abstract

Proton exchange membrane fuel cells (PEMFCs) have been widely studied as an efficient and environmentally friendly energy conversion technology in recent years. However, the high cost, easy poisoning and complex synthesis methods of noble metal catalysts have hindered their commercialization. Therefore, in this paper, a non-noble metal composite catalyst CuFeCo/C for the oxygen reduction reaction (ORR) was prepared by using a facile liquid-phase reduction method. The ORR kinetic performance of CuFeCo/C was evaluated by cyclic voltammetry (CV), linear sweep voltammetry (LSV) and rotating ring-disk electrode (RRDE) tests. The results show that the oxygen reduction peak of CuFeCo/C appears at about 0.64 V, the half-wave potential is about 0.73 V, the limiting current density is about −16.51 A·m^−2^, and the Tafel slope is about −0.08. The 10,800 s chronoamperometry test shows that the catalyst has a very good long-term cycle stability. This indicates that the CuFeCo/C composite catalyst has strong stability, good conductivity and ORR catalytic activity under alkaline conditions, which can promote the large-scale commercial application of PEMFCs.

## 1. Introduction

PEMFC is one of the important ways to utilize hydrogen energy. Widespread use of PEMFC as a power device is an effective way to reduce carbon emissions, improve the environment and address global warming [1,2]. However, the ORR at the cathode of PEMFC is very slow. To improve its working efficiency, a large amount of platinum-based catalysts are needed. The dependence on noble metals leads to a high cost of PEMFC, which is one of the main factors restricting the commercial application of PEMFC [3,4,5]. In addition, the lifespan issue of PEMFC is also a major bottleneck for its commercialization [6,7,8]. Therefore, developing efficient and stable ORR catalysts is an effective way to reduce the cost of the battery [9,10]. Research on PEMFC catalysts mainly focuses on two aspects: one is to study low-platinum [11,12] or platinum alloys [13,14,15], and find methods and technologies to reduce the amount of platinum without excessive loss of its efficiency; the other is to find materials to replace platinum [16,17]. Both of these research methods aim to reduce the cost of PEMFC without losing its catalytic efficiency, and to enhance its broader application value. The former is an effective way to promote the commercialization of PEMFC. However, if PEMFC is to be further promoted, an important measure is to develop catalysts with much higher performance and lower cost. Due to the synergistic effect between metals, multi (binary and ternary)-metal catalysts show better ORR performance and have attracted extensive attention from researchers [18,19]. Because there is a strong synergistic effect among ternary metals, which exhibit superior ORR catalytic activity. Therefore, studying catalysts for ORR-based on ternary metals is of great significance for improving the performance of PEMFC. Considering the cost, developing multi-metal non-noble metal composite catalysts for ORR is crucial for reducing the cost of PEMFC and improving their efficiency.

In recent years, with the development of technology and the deepening of research, transition metals (mainly copper, iron, cobalt, etc.) and their composite materials have been favored by researchers due to their wide availability, large reserves, low cost and good stability. The use of these non-noble metals combined with carbon to replace platinum has become one of the hot research topic for PEMFC cathode catalysts [20,21,22,23,24,25]. These catalysts are usually composed of conductive supports and transition metals. Conductive support materials can provide electronic conductivity, while transition metals can enhance the ORR activity of the catalyst. Among numerous support materials, carbon materials are used as the support due to their excellent electrical conductivity [26,27,28]. By combining non-noble metals with functionalized structures and carbon support, the catalytic performance of the composite materials can be effectively improved [29]. In recent years, many experts and scholars at home and abroad have conducted extensive research on the performance of these transition metal composite catalysts. Through various combinations and modifications of materials, many high-performance non-noble metal composite catalysts have been successfully developed [19]. However, the synthesis method of the catalyst is relatively complex, which is not conducive to the large-scale commercial application of PEMFC.

In view of this, this paper aims to study the performance of multi-metal non-noble metal composite catalysts in ORR. Among numerous non-noble transition metals, Fe can efficiently cleave O-O bonds. After the introduction of Cu, Cu and Fe dual sites can accelerate O-O cleavage, optimize Fe site activity through electronic regulation and enhance catalytic performance in alkaline environments [30]. Co is more active in ORR due to its stronger oxygen activation ability, stable high valence state and d-orbital electron arrangement. However, Ni relies on composite structures or doping to optimize active sites, resulting in weaker activity in ORR [31]. Therefore, in this paper, it is planned to prepare a highly efficient ternary non-noble metal catalyst (CuFeCo/C) through a facile liquid-phase reduction method. This is of great significance for promoting the commercialization process of PEMFC and reducing battery costs. At the same time, it also provides data support and basis for the research of multi-metal non-noble metal composite catalysts in PEMFC.

## 2. Experimental Section

### 2.1. Preparation Process of CuFeCo/C

In this paper, CuFeCo/C composite catalyst was prepared by a facile liquid-phase reduction method using copper salt (copper(II) chloride dihydrate, AR, Macklin, Shanghai, China), iron salt (ferrous sulfate heptahydrate, AR, Macklin, Shanghai, China), cobalt salt (cobalt(II) chloride hexahydrate, AR, Macklin, Shanghai, China) and activated carbon powder (AR, Sinopharm, Beijing, China) as raw materials. Firstly, 789.5 mg of activated carbon powder was added to a clean beaker, then 100 mL of distilled water was added and ultrasonicated for 10 min to obtain an activated carbon powder suspension. Then, 320.0 mg of cobalt(II) chloride hexahydrate, 687.9 mg of copper(II) chloride dihydrate and 2243.5 mg of ferrous sulfate heptahydrate were, respectively, added to the activated carbon powder suspension, followed by ultrasonication for 10 min. The molar ratio of copper, iron and cobalt is 3:6:1. Finally, in order to ensure the complete reduction of metal ions to elemental metal, sufficient reaction is required. Sodium borohydride needs to be in excess, which is 1.4 times the total moles of metal salts. Thus, 712.3 mg of sodium borohydride (AR, Damao, Tianjin, China, to ensure complete reduction in these metal ions) was added to another dry and clean beaker. The metal salt solution obtained in the previous step was rapidly dropped into the beaker containing sodium borohydride using a constant pressure dropping funnel (with a dropwise addition rate of 50 mL/min), and stirred with an electric stirrer (200 rpm). Bubbles could be observed. When the bubbles stopped, it indicated that the metal ions were completely reduced. The product was washed with water three times and then with ethanol (AR, Sinopharm, Beijing, China) for three times, and finally dried in a vacuum drying oven at 60 °C for 24 h to obtain the CuFeCo/C composite catalyst. CuFeCo catalyst was prepared using the same method, except for the absence of carbon powder.

### 2.2. Characterization

The phase analysis of the sample was carried out on a X-ray powder diffractometer (D8 ADVANCE, Bruker AXS, Karlsruhe, Germany). The surface microstructure of the sample was tested using a field-emission scanning electron microscope (SEM, ZEISS Sigma 500, Carl Zeiss, Oberkochen, Germany) equipped with energy-dispersive X-ray (EDX) detector. X-ray photoelectron spectroscopic (XPS) measurement was conducted on an Axis Ultra instrument from Kratos using monochromatic Al Kα radiation. Raman scattering spectra were recorded on a laser Raman microscope system (Horiba LabRAM HR Evolution, Horiba, Kyoto, Japan). FTIR spectra was carried out on a Fourier transform infrared spectrometer (Thermo Nicolet iS 5 FT-IR, Thermo Fisher Scientific, Waltham, MA, USA).

### 2.3. Electrochemical Measurement

All electrochemical measurements were carried out using a standard three-electrode configuration on a electrochemical workstation (AUTOLAB PGSTAT302N, Metrohm AG, Herisau, Switzerland), where the Ag/AgCl (KCl-saturated) electrode and a platinum sheet rod were used as reference and counter electrode, respectively. To ensure the repeatability of the experiment, the working electrode for each catalyst was prepared under uniform conditions. The procedure for the preparation of a working electrode was as following: the catalyst powder (5 mg) was dispersed in 800 µL of ethanol with 40 µL of Nafion solution (5 wt %, Sigma-Aldrich, St. Louis, MO, USA) under sonication to obtain a homogeneous suspension. Then, the catalyst ink (10 μL, 0.30 mg·cm^−2^) was dropped on the working electrode surface. For ORR tests, cyclic voltammetry (CV) curves were collected in a N_2_-saturated or O_2_-saturated 0.1 M KOH electrolyte at a scan rate of 20 mV·s^−1^ (from −0.8 to 0.2 V). Additionally, the activity for ORR was also evaluated by LSV at a scan rate of 10 mV·s^−1^ (from −0.5 to 0.2 V) in O_2_-saturated 0.1 M KOH electrolyte with different rotation speeds of 400 rpm, 625 rpm, 900 rpm, 1225 rpm, 1600 rpm and 2025 rpm, respectively. The ORR stability in O_2_-saturated 0.1 M KOH solution was tested by current versus time (i-t) test with a rotating speed of 1600 rpm.

## 3. Results and Discussion

### 3.1. Characterization of CuFeCo/C

**Figure 1:**
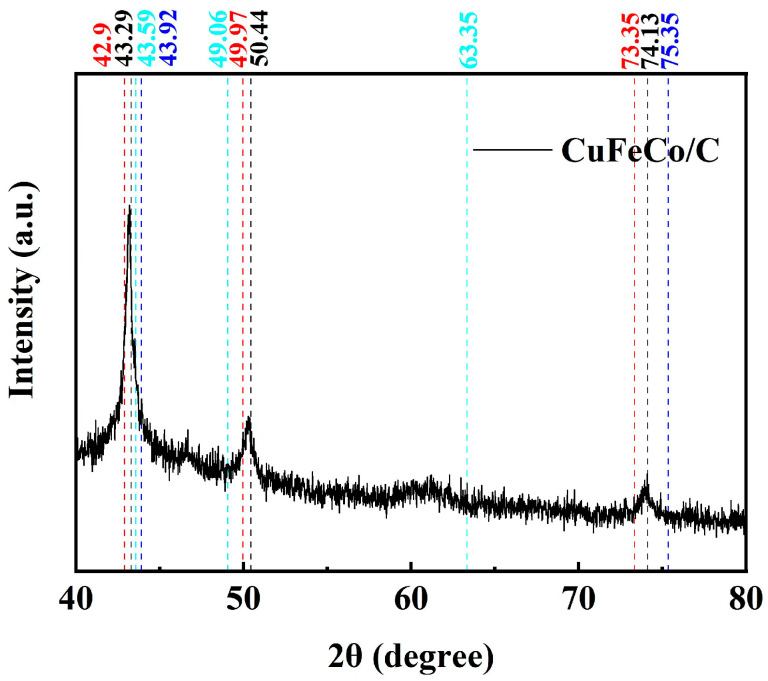
XRD patterns of CuFeCo/C (40 kV, 40 mA, 0.100 steps, continuous PSD fast, coupled TwoTheta).

The XRD pattern of CuFeCo/C is shown in Figure 1. From the analysis of the figure, the diffraction peaks at 2θ of 43.29°, 50.44°and 74.13° are consistent with the characteristic peaks of standard card 4-836, which confirms that the prepared sample contains Cu. The diffraction peaks with diffraction angles 2θ of 42.90°, 49.97°and 73.35° are consistent with the characteristic peaks of standard card 65-4150, which proves the existence of Fe. The diffraction peaks at 2θ of 43.59°, 49.06°and 63.35° are consistent with the characteristic peaks of standard card 47-1896, indicating the existence of Co in the composite catalyst. The diffraction peaks with diffraction angles 2θ of 43.92° and 75.30° are consistent with the characteristic peaks of standard card 6-675, which proves that there is C in the composite catalyst. Therefore, based on the above analysis, it can be concluded that we have successfully prepared a composite catalytic material of CuFeCo/C.

**Figure 2:**
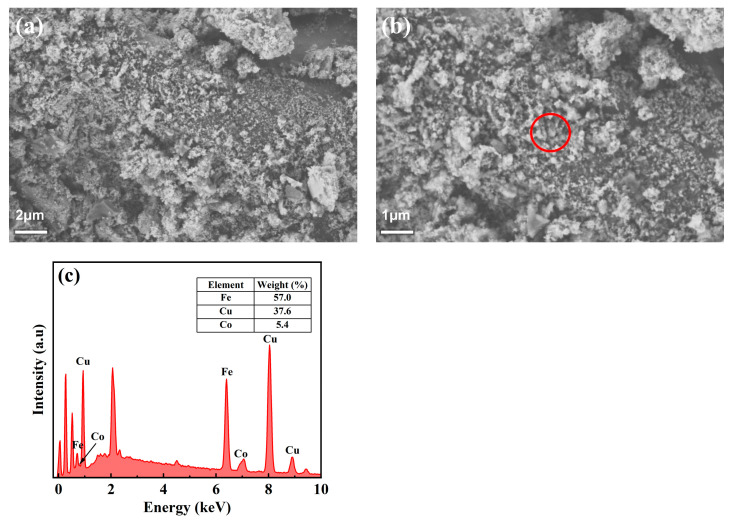
(**a**,**b**) SEM images of CuFeCo/C at different magnifications and (**c**) EDX of CuFeCo/C.

The microstructure of the prepared CuFeCo/C catalyst was investigated by SEM. The morphology of the catalyst at different magnifications is shown in Figure 2a,b. It can be observed from the figure that the prepared CuFeCo/C catalyst is a black, powder-like solid, with CuFeCo attached to the surface of activated carbon. The particle size is approximately between 100 nm and 500 nm. EDX was conducted at the circular position in Figure 2b. And the mass composition of elements in the composite catalyst CuFeCo/C is shown in Figure 2c. According to calculations, it is known that the molar ratio (Cu:Fe:Co) is about 3.5:6:0.5, which is close to the theoretical value (3:6:1). It indicates the successful preparation of the catalyst.

**Figure 3:**
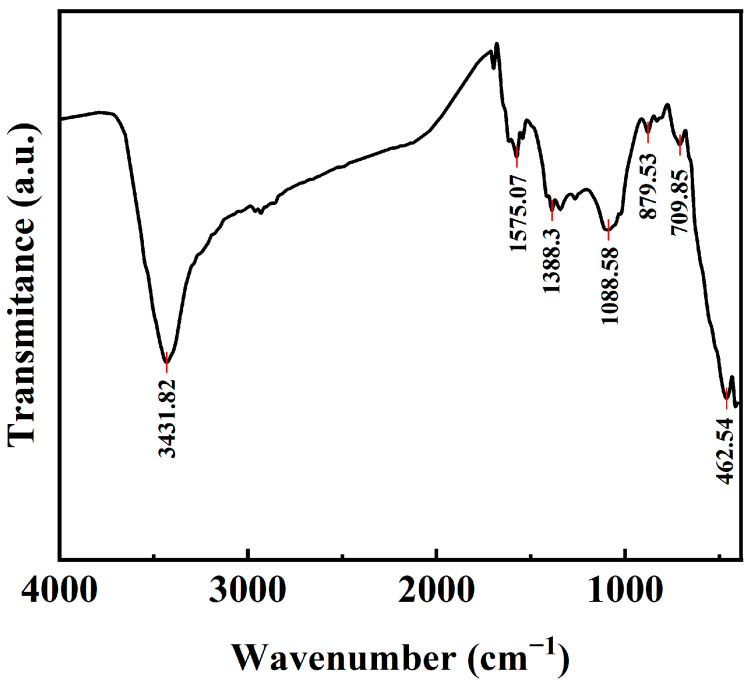
FTIR spectra of CuFeCo/C.

FTIR spectra detection results are shown in Figure 3. The peak at 3431.82 cm^−1^ originates from the stretching vibration of O-H, while the telescopic vibration of the C=C (sp^2^) bond produces a peak at 1575.07 cm^−1^. The bending vibration of C-H is indicated by the absorption peak at 1388.3 cm^−1^. The stretching vibration of C-O occurs at 1088 cm^−1^. The peaks at 879.53 cm^−1^ and 709.85 cm^−1^ may be attributed to the out-of-plane bending vibration of C-H. The presence of these oxygen-containing functional groups in the CuFeCo/C composite material enhances the affinity of CuFeCo with the carbon support, which can provide abundant metal nucleation sites, allowing the metal to disperse better on the support, exposing more catalytic active sites and thus improving the activity of the catalyst [32].

**Figure 4:**
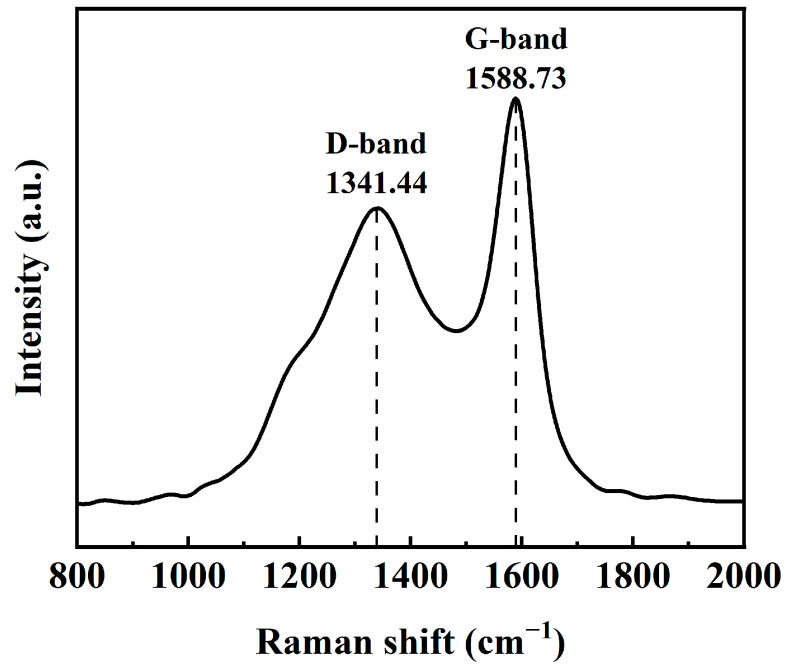
Raman spectra of CuFeCo/C.

Raman spectra (Figure 4) manifests that CuFeCo/C has characteristic peaks, which appear at 1341.44 cm^−1^ (D band) and 1588.73 cm^−1^ (G band). The D-band represents the vibration of the graphite structure induced by the defect, characterizing the disorder or defect density, while the G-band corresponds to the in-plane expansion vibration of the sp^2^ hybrid carbon atoms, reflecting the degree of graphitization. Research shows that the lower the D/G band-intensity ratio (I_D_/I_G_), the higher the graphitization degree [33]. It is obvious that the I_D_/I_G_ (0.74) is low in CuFeCo/C, which indicates that the composite catalyst has a large number of defects and more catalytic active sites.

**Figure 5:**
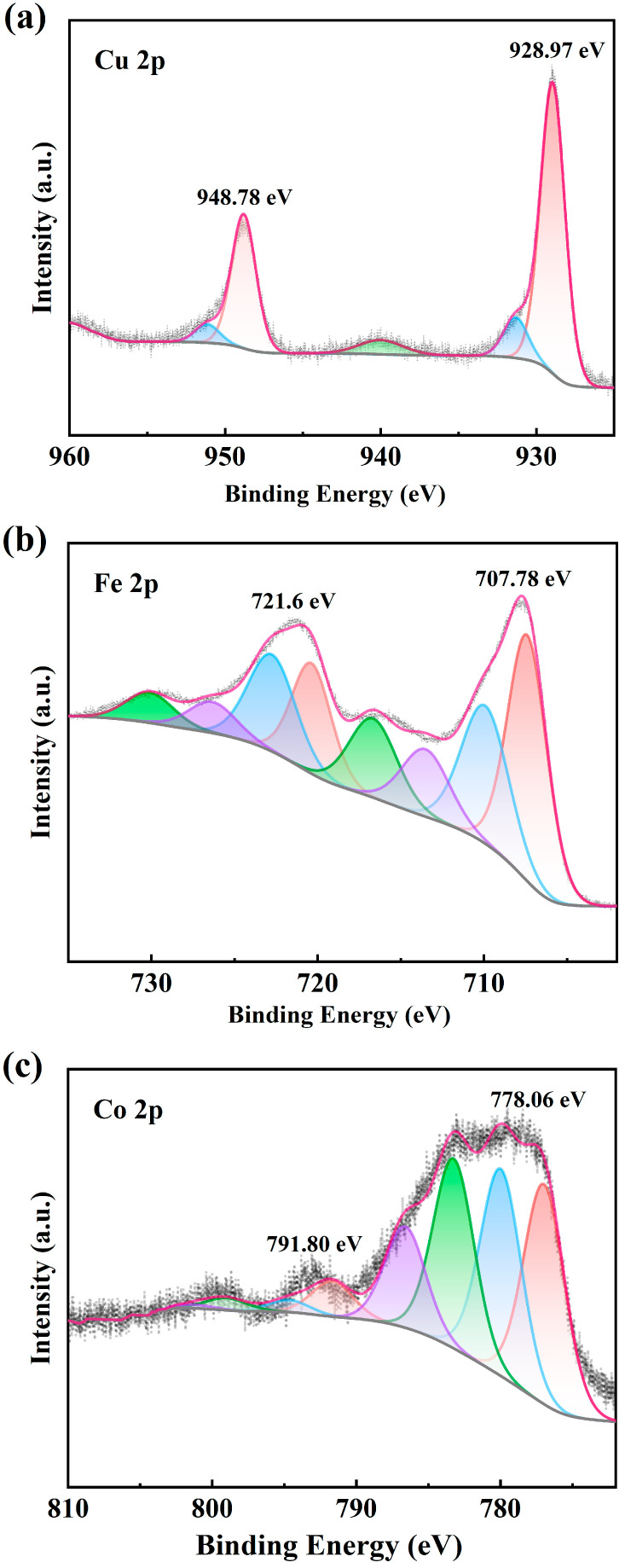
XPS spectra of (**a**) Cu 2p, (**b**) Fe 2p, and (**c**) Co 2p levels of CuFeCo/C.

XPS measurement of CuFeCo/C was used to characterize the chemical valence states (Figure 5). In the Cu 2p spectra for CuFeCo/C (Figure 5a), the peak at 928.97 eV belongs to Cu in metallic state, while the peak at 948.78 eV originates from Cu-O species [34]. From the Fe 2p spectra (Figure 5b), the peaks at 707.78 eV and 721.6 eV both indicate that Fe exists in a metallic state [35]. The valence state of Co can be analyzed from Figure 5c. The valence state of Co can be analyzed from Figure 5c. The main peak near 778.06 eV indicates that Co mainly exists in the form of zero valence, and there is a weak small peak at 791.80 eV, indicating that a small amount of Co exists in the form of Co-O species [33]. From the analysis, it can be concluded that the ternary components in the composite catalyst CuFeCo/C mainly exist in the form of metallic states.

### 3.2. Electrochemical Properties of CuFeCo/C

**Figure 6:**
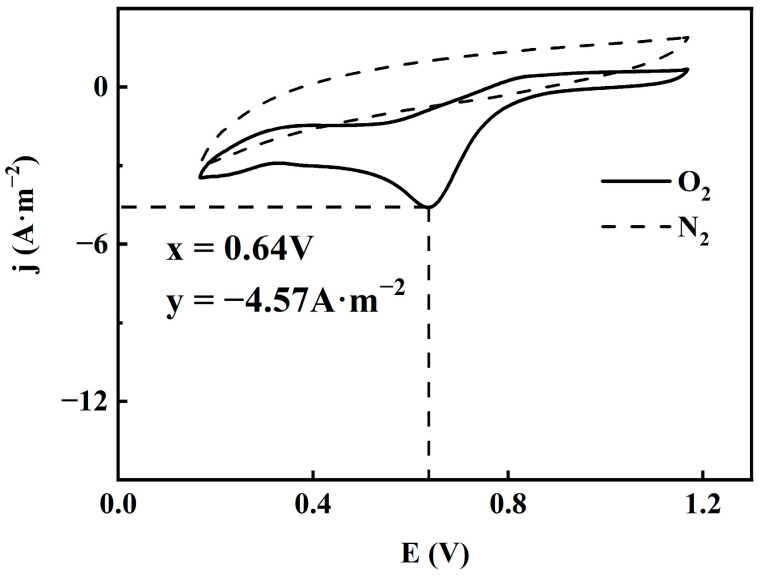
CV curve of CuFeCo/C in N_2_ or O_2_ saturated state.

In order to analyze the catalytic performance of CuFeCo/C, the ORR electrochemical performance of the prepared catalyst was tested by using a three-electrode system. The CV curve of the prepared CuFeCo/C catalyst in 0.1 M KOH electrolyte solution under N_2_ or O_2_ saturation is shown in Figure 6. The dotted line in the figure is the CV curve under N_2_ saturation, and the solid line is the CV curve under O_2_ saturation. The horizontal coordinate is the voltage and the vertical coordinate is the current density. It can be observed from Figure 6 that CuFeCo/C catalyst has no oxygen reduction peak in the electrolyte saturated with N_2_, and the CV curve is a closed approximately elliptical shape without oxygen reduction peak. In the O_2_ saturated electrolyte, there is a significant oxygen reduction peak (0.64 V), and the corresponding current density is −4.57 A·m^−2^. The more positive the potential of oxygen reduction peak and the higher the current density, the better the ORR performance of the prepared catalyst [36], so CuFeCo/C exhibits strong catalytic activity.

**Figure 7:**
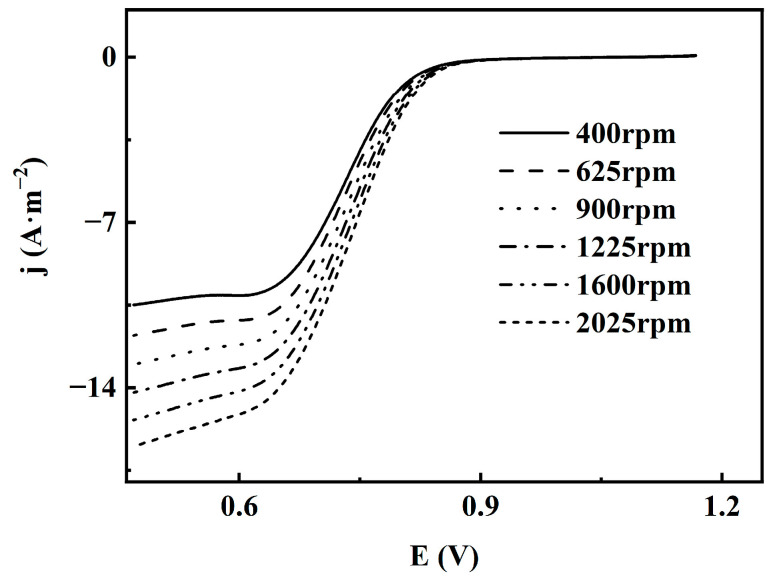
LSV curves of CuFeCo/C at different rotational speeds.

To further investigate the activity of the catalyst, LSV tests were conducted on CuFeCo/C. The LSV curves of CuFeCo/C at different rotation speeds are shown in Figure 7, with voltage on the *x*-axis and current density on the *y*-axis. The curves from top to bottom correspond to rotation speeds of 400 rpm, 625 rpm, 900 rpm, 1225 rpm, 1600 rpm and 2025 rpm, respectively. It is indicated that the corresponding limiting current densities at different rotation speeds are −10.49 A·m^−2^, −11.79 A·m^−2^, 13.02 A·m^−2^, −14.20 A·m^−2^, −15.36 A·m^−2^ and −16.51 A·m^−2^, respectively. It suggests that as the rotation speed increases, the absolute value of the limiting current density also increases, showing a positive correlation. However, the half-wave potential remains relatively stable at around 0.73 V. The improvement of catalytic activity may be closely related to the synergistic effect between metals in ternary systems [19,24,25].

**Figure 8:**
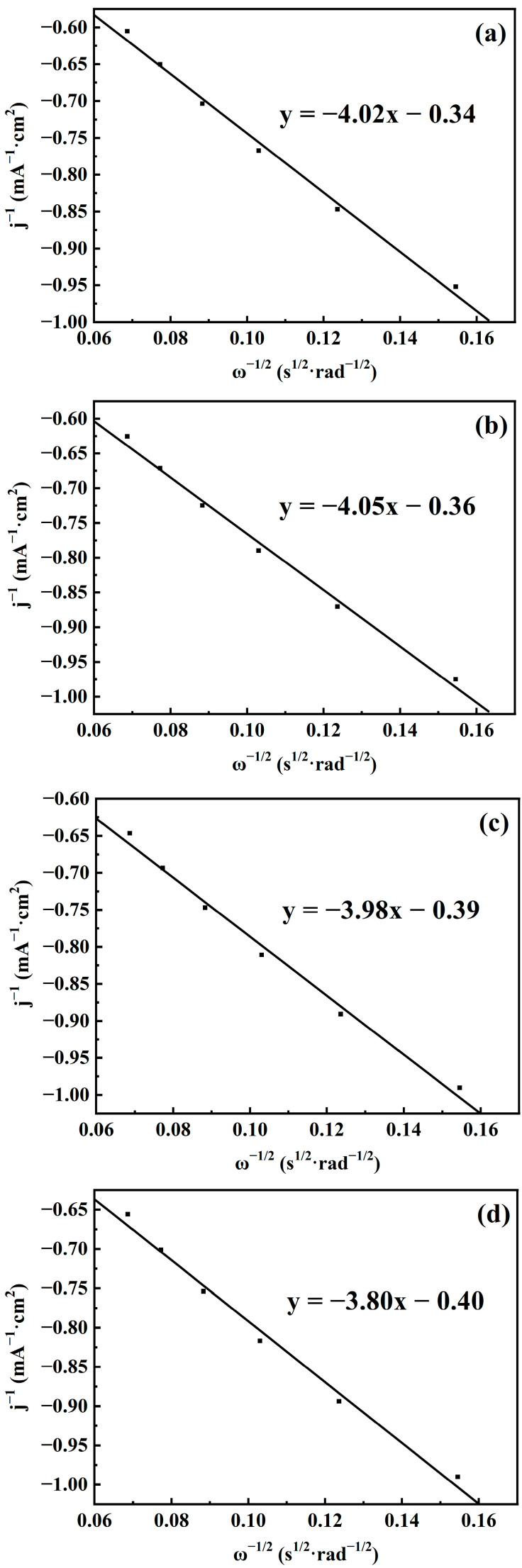
K-L fitting curve of CuFeCo/C at (**a**) 0.47 V, (**b**) 0.52 V, (**c**) 0.57 V, and (**d**) 0.59 V, respectively.

To determine whether the electron transfer in the ORR catalyzed by CuFeCo/C follows a 4-electron or 2-electron transfer pathway, K-L fitting was conducted on the LSV curves of CuFeCo/C with different rotation rates at voltage values of 0.47 V, 0.52 V, 0.57 V and 0.59 V, respectively. The fitting curves are shown in Figure 8. As can be seen from the figure, each fitting curve has a good fitting degree, and the absolute values of the slopes are 4.02, 4.05, 3.98 and 3.8, respectively. According to the slopes of the fitting curves and using the K-L equation (Equations (1) and (2)), the number of transferred electrons can be obtained. After calculation, the number of transferred electrons is 2.29, 2.28, 2.32 and 2.43, respectively. Therefore, the ORR mainly follows a 2-electron transfer pathway, where O_2_ is first reduced to H_2_O_2_ and then to H_2_O.

(1) $$\frac{1}{j}=\frac{1}{j_{k}}+\frac{1}{j_{L}}=\frac{1}{{B\;\omega }^{1/2}}+\frac{1}{j_{k}}$$

(2) $$B=0.62\;\mathrm{nF}{\left(D_{O}\right)}^{2/3}\nu^{-1/6}C_{O}$$

**Figure 9:**
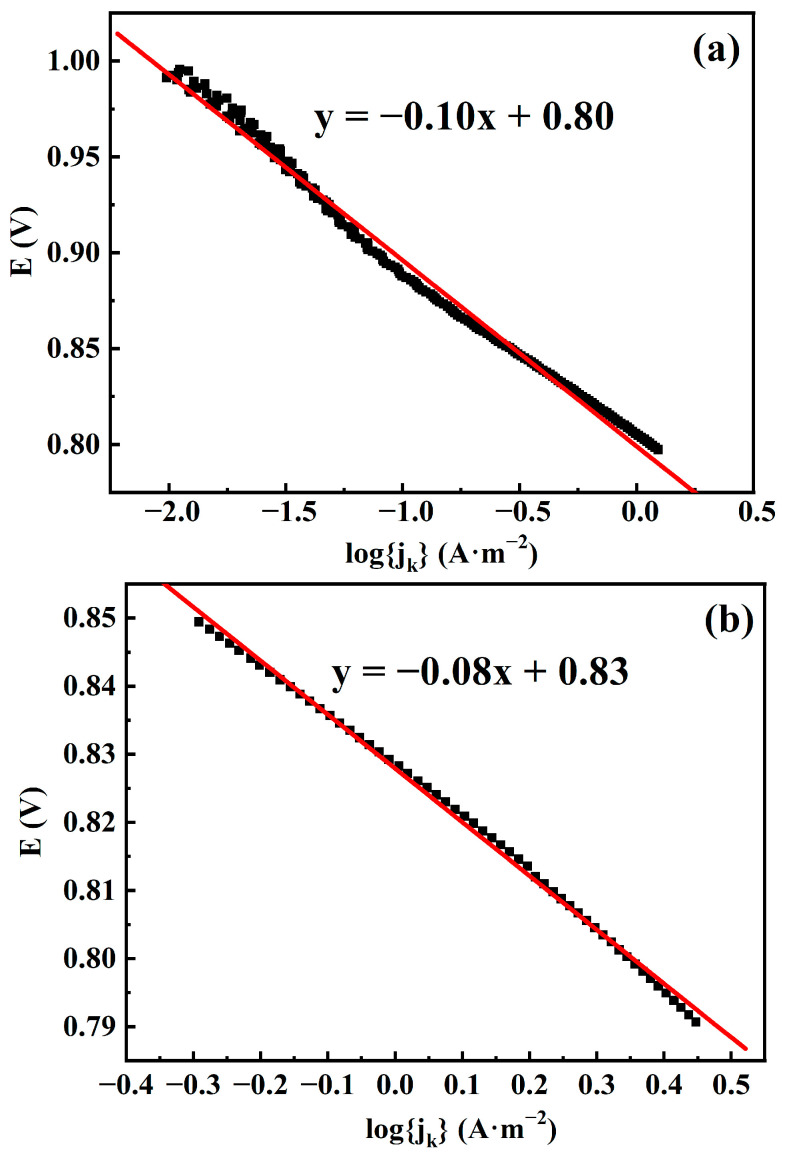
Tafel curve of (**a**) CuFeCo and (**b**) CuFeCo/C at 1600 rpm. Black: experimental data; Red: linear fit.

In order to investigate the kinetic performance of the prepared CuFeCo/C catalyst in detail, it is analyzed with the help of Tafel slope in this paper. Based on the LSV curve at 1600 rpm, the Tafel curves of CuFeCo and CuFeCo/C are shown in Figure 9 and the fitted Tafel slope is about −0.10 and −0.08, respectively. Compared with CuFeCo, CuFeCo/C has lower absolute value of Tafel slope and better kinetic performance. This is mainly attributed to the better synergistic effect between Cu, Fe, Co and good electrical conductivity of carbon powder [19]. At the time, the porous structure of activated carbon is beneficial to the dispersion of CuFeCo, increasing its specific surface area and the number of catalytic active sites [28].

**Figure 10:**
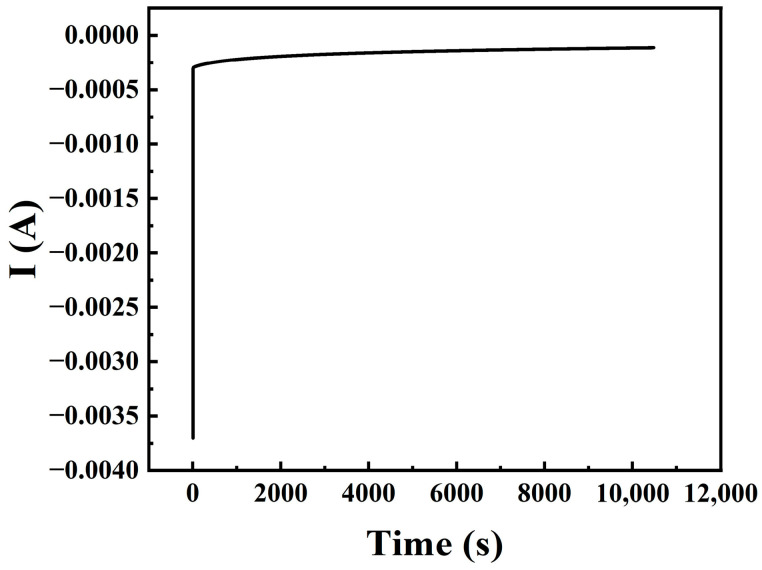
Current versus time test curve of catalyst CuFeCo/C in 0.1 M KOH saturated aqueous solution with oxygen for 10,800 s.

Stability is an important indicator for evaluating the ORR activity of a catalyst. In this paper, the stability of the CuFeCo/C catalyst was investigated by chronoamperometry. The electrode rotation speed was set at 1600 rpm, and the catalyst was subjected to a 10,800 s current versus time test in 0.1 M KOH electrolyte saturated with O_2_. The results are shown in Figure 10. It can be seen from the figure that the current versus time curve tends to a stable straight line, indicating that the CuFeCo/C catalyst is relatively stable during the 10,800 s operation, with almost no current decay. This suggests that the catalytic active substances of CuFeCo/C are not easily lost and it is a stable cathode multi-component non-noble metal catalyst.

**Figure 11:**
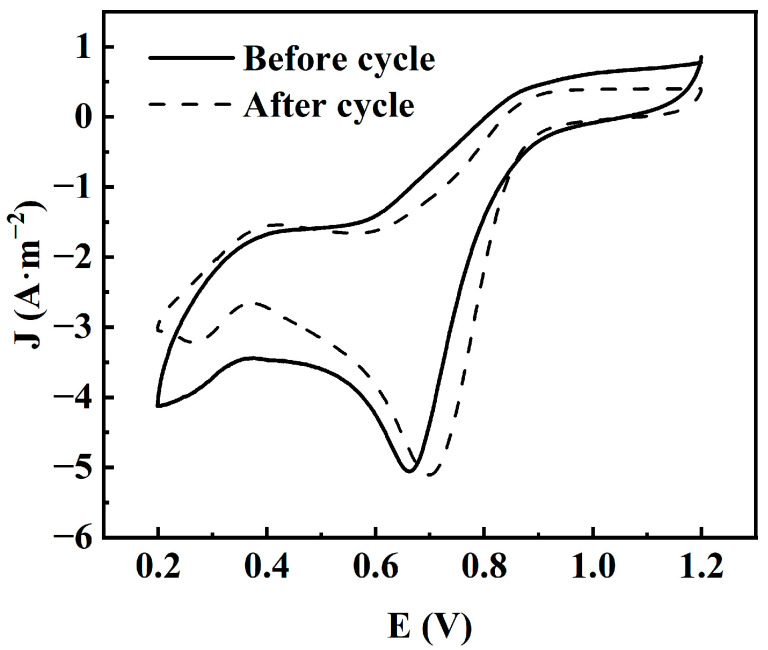
CV curve of CuFeCo/C before and after the cycle.

The electrochemical stability of the catalyst can be further evaluated by comparing the CV curves before and after cycling. As shown in Figure 11, after the 10,800 s current versus time test, the CV curve of the catalyst changes very little compared with that before cycling, and the oxygen reduction peak potential remains almost unchanged. This further confirms that the CuFeCo/C catalyst has a very good long-term cycle stability.

## 4. Conclusions

To sum up, a non-noble metal composite catalyst CuFeCo/C for ORR was prepared by a facile liquid-phase reduction method using activated carbon as the support, with a particle size of 100–500 nm. The research shows that the half-wave potential of the catalyst in ORR is stable at 0.73 V, and the limiting current density is −16.51 A·m^−2^ (rotation speeds of 2025 rpm). The ORR path is mainly a two-electron transfer process. The prepared CuFeCo/C has good ORR performance (especially the very good long-term cycle stability), which can be attributed to the synergistic effect among Cu, Fe and Co and the enhanced electronic interaction between metal and carbon support. This provides a facile and effective idea for the synthesis of non-noble metal catalysts for the cathode of PEMFC, and also provides strong support for subsequent commercial application of such catalysts.

## Data Availability

The original contributions presented in this study are included in the article. Further inquiries can be directed to the corresponding author.
